# Accelerator mass spectrometry: an analytical tool with applications for a sustainable society

**DOI:** 10.1140/epjti/s40485-023-00088-3

**Published:** 2023-03-24

**Authors:** William E. Kieser

**Affiliations:** Department of Physics and A. E. Lalonde AMS Laboratory, University of Ottawa, Ottawa, K1N 6N5 Canada

## Abstract

Accelerator Mass Spectrometry (AMS) adds the techniques of higher energy charged particle acceleration to the basic principles of Isotope Ratio Mass Spectrometry (IRMS) to provide extremely low detection capability (below 1 femtogram) of rare isotopes in samples of natural materials as small as 1 mg. Depending on the element selected and the configuration of the equipment, rare isotope sensitivities can reach less than one part in 10^15^. The advantages of this small sample size and high sensitivity for the detection of rare isotopes include a) the economic benefit of collecting, shipping and preparing much smaller samples, and b) the ability to analyse specific chemical compounds within the sample. For the latter advantage, the pathway taken by that compound through a complex system can be more precisely traced or, in the case of radioactive isotopes, more precise chronological information can be provided. The paper is an amplification of material which was presented at the IAEA International Conference on Accelerators for Research and Sustainable Development: novel concepts and technical innovation. It begins with a basic overview of AMS technology, with an emphasis on how the use of higher energy contributes to this enhanced sensitivity, and then provides several examples of new AMS technologies which reduce the energy and space requirements for such systems. Several examples of applications which contribute to the investigation of sustainability in other areas of environmental concern are then briefly described.

## Introduction

### Requirements leading to the development of AMS

The use of accelerators to increase the sensitivity of mass spectrometry has had a long history, dating back to 1939 when Alvarez and Cornog used the recently developed Berkeley cyclotron to identify the existence of ^3^He in a sample of ^4^He at a ratio of ^3^He/^4^He ≈ 1 × 10^−6^ [1]. However, such measurements remained isolated experiments until the development of tandem electrostatic accelerators [2] and the negative ion sources needed to provide anions (negative ions) from solid, rather than only gas samples [3]. During this time, the technique for measuring the age of organic samples was developed by Libby [4]. He counted the *β* particles emitted in the radioactive decay of ^14^C in a sample and corelated this count rate with the age of the sample, using a series of known-age samples for calibration. This technique rapidly gained widespread use in the fields of Earth and environmental sciences, as well as in Archaeometry; the half-life of ^14^C (5730 a) permitted good chronological measurements for late Quaternary geological events and for the development of human societies. By the mid 1970s, the demand for precise and rapid ^14^C dating was exceeding the capacity of the decay-counting method, even with improvements of newer technology, such as liquid scintillation counting. Mass spectrometry at this time was still hindered by the overwhelming presence of the isobar ^14^N which has very nearly the same mass ($\Delta \mathrm{M}/\mathrm{M} = 3.06 \times 10^{-4}$). This barrier was overcome in 1977 by Purser et al. [5], who showed that the negative ions (needed for injection into tandem electrostatic accelerators) of ^14^N are unstable, whereas those of ^14^C are relatively robust. This was followed quickly by several papers demonstrating the use of tandem electrostatic accelerators, equipped with Middleton type caesium sputter ion sources [3], to provide ^14^C measurements with acceptable accuracy [6, 7]. The two key technical requirements: ion energy provided by the tandem accelerator and high ion currents from the sputter source were now in place to take advantage of the fortuitous instability of the ^14^N anion.

### Expansion to applications beyond ^14^C

Following this initial discovery, the number of isotopes which could easily be analysed expanded rapidly to include ^10^Be [8, 9], ^36^Cl [10] and ^129^I [11]. Within another decade, the actinides were included [12], for example, a less abundant isotope ^236^U [13]. In the over 40 years of the availability of AMS analyses, many applications in Earth, environmental, planetary, biomedical and cultural sciences have been developed. Of particular interest to sustainability are the contributions made to climate change research using ^14^C and more recently ^26^Al, ^36^Cl and ^10^Be, to provide details about previous climate change events and to monitor the specific events associated with current changes, such as permafrost thawing, sources of atmospheric methane and carbon cycling in the oceans. For the energy sector, atmospheric measurements of ^14^C are used to assess the efficacy of bio-remediation programs for fossil fuel spills and the actinides and fission fragments are analysed to monitor the production, use and disposal of nuclear fuel and related material. Cultural applications include collaborations with indigenous communities to provide chronologies for events chronicled in their oral histories, some of which include their adaptation to earlier environmental changes [14].

### AMS and sustainability

There are currently approximately 160 AMS systems in operation throughout the world, ranging in acceleration voltage from 200 kV to 15 MV, a number which has doubled in the past 10 years. However, as the space required and the cost of such equipment scales with the terminal voltage, manufacturers and many of their clients are opting for smaller systems. Those which operate at the lowest of these voltages can be specifically designed for one element (typically carbon isotopes), which permits the use of permanent, instead of electro-magnets—a significant saving in installation and operating costs. In addition, there are several multi-element machines coming on line which operate at 300 kV. In both cases, these systems can be equipped with integrated sample preparation equipment, such as elemental analysers, carbonate analysis systems or even IRMS systems for abundant stable isotope analyses of the same sample—a further economy in labour and time.

## Techniques which enable AMS sensitivity for rare isotopes

### Challenges and solutions

**Figure 1:**
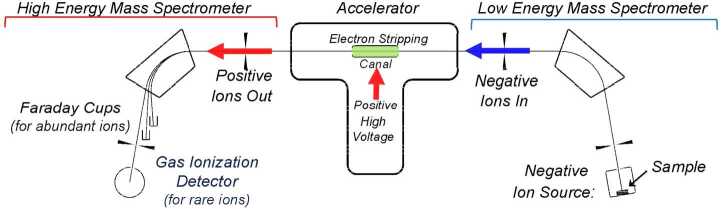
Basic parts of an AMS system using a tandem accelerator.
- The low concentration of the rare isotope of interest, as well as the increasingly smaller samples that need analysis, require an ion source which can efficiently generate high currents. For example, whereas an IRMS instrument can provide precise results with a nano-ampere beam of analyte ions, an AMS source requires 10 s to 100 s of micro-amperes to provide a sufficient number of rare isotope anions to achieve statistical precisions of between 1% and 1‰. These are typically sources which sputter solid samples with a high current of Cs” ions, but some systems are now using compact microwave or electron cyclotron resonance (ECR) sources with larger currents, albeit of positive ions, and charge changing them to anions where necessary [15].

The requirement to measure isotope ratios at very low concentrations (typically 1 part in 10^12^–10^15^) sets AMS apart from IRMS in several ways:

- At the typical concentration of the rare isotope, the measurement can be compromised by an increased number of isobars (atoms or molecules of a different element which have a mass so close to the mass of the rare isotope of interest that they are not resolvable by conventional mass analysis techniques, such as magnet-electric analyzer combinations or radio-frequency quadrupoles (RFQs). The initial solution provided by AMS, as described in Sect. 1.1, is the use of negative ions. However, this solution provides only a few, albeit important, solutions—^14^C (^14^N), ^26^Al (^26^Mg) and ^129^I (^129^Xe). For molecular isobars, the accelerator provides the energy necessary to break up the molecules in the accelerator terminal electron stripping canal (see Fig. 1). For other atomic isobars, the accelerator provides the energy required to use energy loss methods such as energy degrader foils or gas-filled magnets. These, combined with a gas ionization detector, can, with sufficient energy, resolve many isobar issues.

### Basic parts of an AMS system

As most AMS systems still use tandem electrostatic accelerators, the technique will be illustrated using such systems. Some smaller systems use only a single high voltage acceleration gap as indicated in ref. [16], but many of the principles are common to both approaches. The main parts of a tandem accelerator-based system are summarized in Fig. 1.

*The low energy spectrometer section*, which includes the ion source, an electric analyser to select a precise ion energy (if needed) and an injection magnet which selects the ions of a specific momentum. Together these two analyzers define the mass selected for injection. Most systems include a method for rapid switching between the rare and abundant isotopes, usually by accelerating or decelerating either the rare and/or abundant ions before and after the magnet, thus adjusting the ion momentum appropriately and avoiding the slow process needed to switch the field of the injection magnet.

*The accelerator section* includes the high voltage power supply, graded vacuum tubes to conduct the ions to and from the high voltage terminal and an electron stripping device in the terminal. The latter can be either a foil or a windowless gas cell, with a pump to recirculate the stripping gas. These remove electrons from the incoming beam and provides positive ions for further acceleration. With sufficient foil thickness or gas pressure and appropriate ion velocity, molecular ions can be disintegrated into their constituent atoms and the charge state of the positive ions can be optimized to facilitate further analysis.

**Figure 2:**
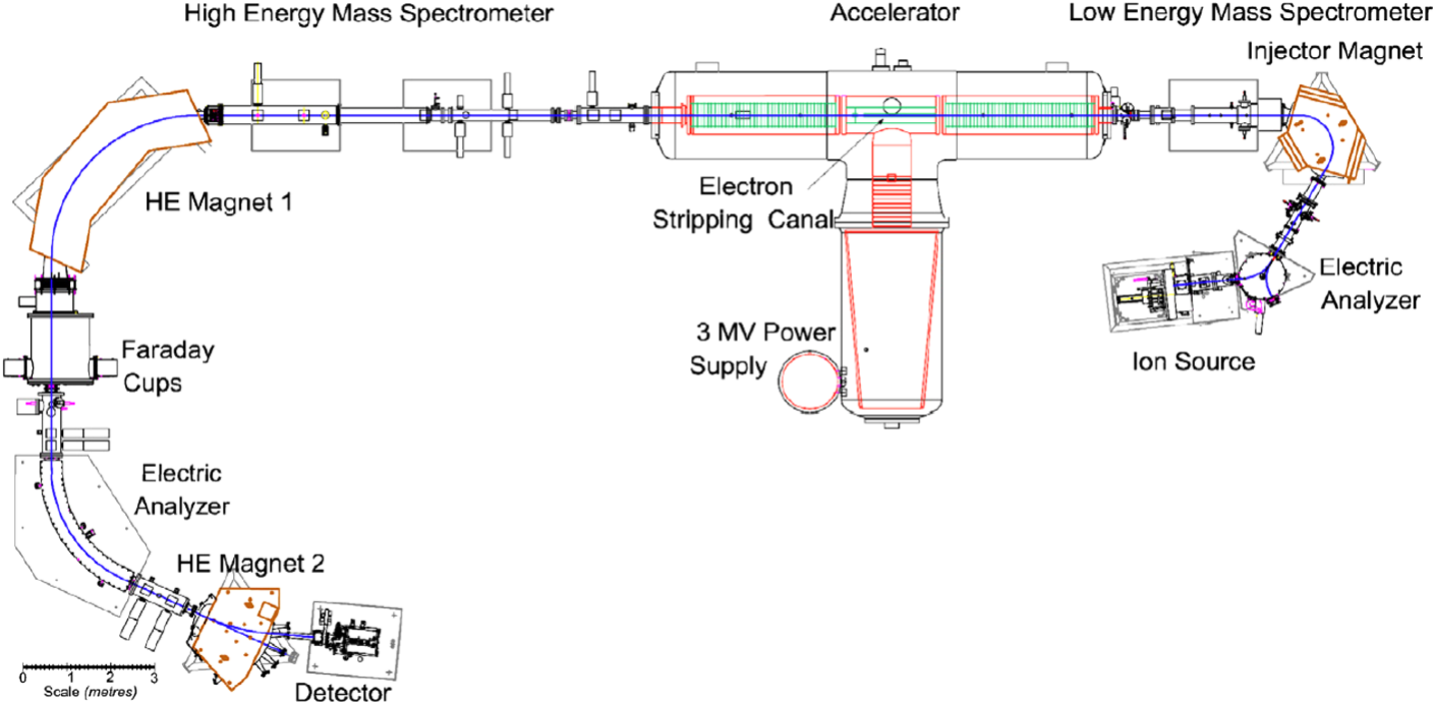
The AMS system at the Lalonde AMS Lab, University of Ottawa. Due to building constraints, the ion beam direction is from right to left

*The high energy spectrometer* section includes the magnets and electric analysers required for the analysis of the positive rare ion beam as well as Faraday cups, usually following the first magnet, to measure the current of the abundant beam(s) and a gas ionization or silicon detector to count the positive rare ions. The gas ionization detector provides extremely low noise, single atom counting as well as energy loss information, which can be useful in further isobar discrimination. A particular implementation of these sections is shown in Fig. 2, a schematic of the AMS system at the Lalonde AMS Lab in Ottawa.

## Sustainability in AMS technology

### Smaller AMS systems

**Figure 3:**
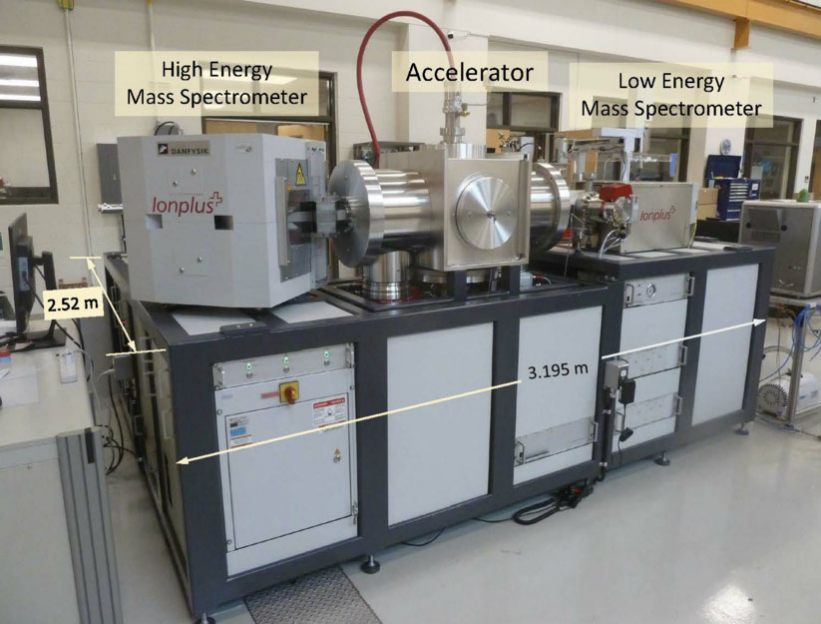
Single Element ^14^C System installed at Lalonde AMS
- Single stage AMS systems in which either the high energy (more usual) or the low energy spectrometer section is placed on a high voltage platform [16, 23]. These systems, with only one short accelerator tube also exhibit enhanced transmission efficiency.

Accelerator manufacturers, in some cases for over a decade, have been building smaller AMS systems, which require significantly less space (a capital cost reduction) as well as less electrical power for their operation. These can be broadly divided into two types:

- Low energy tandem accelerators, using high voltage terminal voltages of between 200 kV (Fig. 3) [17–19] and 300 kV [20–22]. Due to the shorter distance to the accelerator terminal and vacuum tubes with a larger bore, such instruments can provide better transmission efficiency through the accelerator than larger systems.

### Single element AMS systems

**Figure 4:**
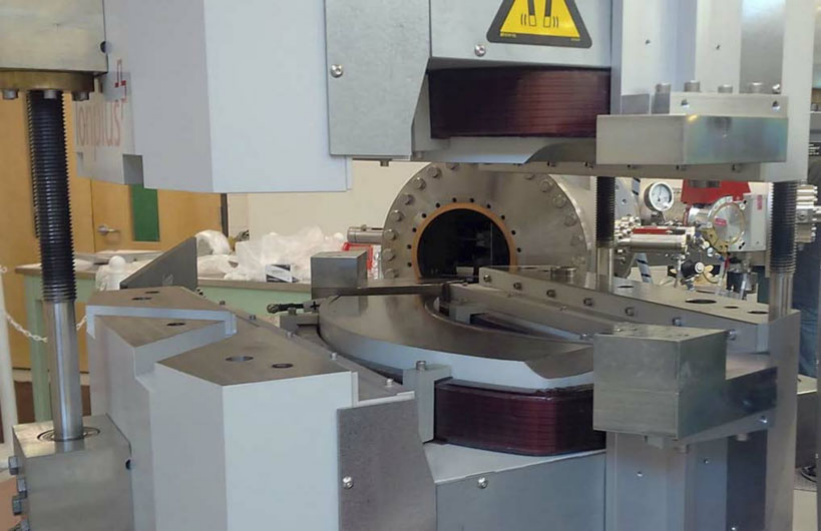
Permanent Magnet in the high energy spectrometer with upper yoke lifted during assembly at Lalonde. Note the small size of the trim coil compared with the space available for a water-cooled full power coil

In larger systems, magnets typically consume the most power (often more than the accelerator). Systems built for specific isotope analysis (e.g. ^14^C), can use permanent magnets with a small electrical coil for minor adjustments (Fig. 4). In addition to conserving electrical power (a typical maximum of 30 W), such magnets eliminate the need for water cooling of the coils, necessary for a conventional electromagnet. In comparison, the 90° high energy magnet shown in Fig. 2 uses ∼1 kW for ^14^C analysis, but up to 16 kW for the actinides. So far, permanent magnets have only been used in the ^14^C system shown in Fig. 3, but this approach could be scaled to other light elements if any of their applications warranted a dedicated single element machine.

An example of a single element system for ^14^C (MICADAS^TM^ Ionplus AG) recently installed at the Lalonde AMS Lab, is shown in Fig. 3 and the high energy permanent magnet in Fig. 4.

## Examples of applications promoting sustainability in other fields

### Carbon in the Arctic environment

It is now well established that the Arctic [24] and the Antarctic [25] regions are experiencing the effects of over a doubling of the current increase in global average temperatures. In terms of carbon storage or emission, this represents a “two-edged sword”, as although the reduced ice cover in the ocean regions permits an increase in biological carbon sequestration in the water column and the sediments, this can easily be overwhelmed by the thawing of permafrost regions with a release of methane as well as carbon dioxide.

AMS carbon isotope analysis has played an important role in the development and testing of models predicting sea-level rise and glacial isostatic adjustment [26], as the timing of the changes since the last glacial maximum (∼20 ka B.P.) matches well with the 5730 a half-life of ^14^C. On-going studies of particulate organic matter in the water column [27] use stable ^13^C and ^15^N measurements as well as ^14^C analysis to indicate the origin of the suspended fraction of this material. DeVernal et al. [28] have used ^14^C analysis of planktonic foraminifera in sediment cores collected along the Lomonosov Ridge to determine the periods of summer open water since the last glacial maximum.

The small sample AMS capability will increase the use of Compound Specific Radiocarbon Analysis (CSRA). The original intention of this approach was to reduce the possibility of heterogeneous sources of ^14^C in samples. A particular compound of interest for climate change is atmospheric methane, which is a much more potent greenhouse gas than CO_2_ and can be derived from many sources. Recent work by Espic et al. [29] shows the difficulty of preparing such samples; a further reduction in sample size requirements will decrease the current logistical costs of obtaining and shipping 100 L sized “bags” of air samples from distant field sites.

### Bio-remediation of hydrocarbon spills

**Figure 5:**
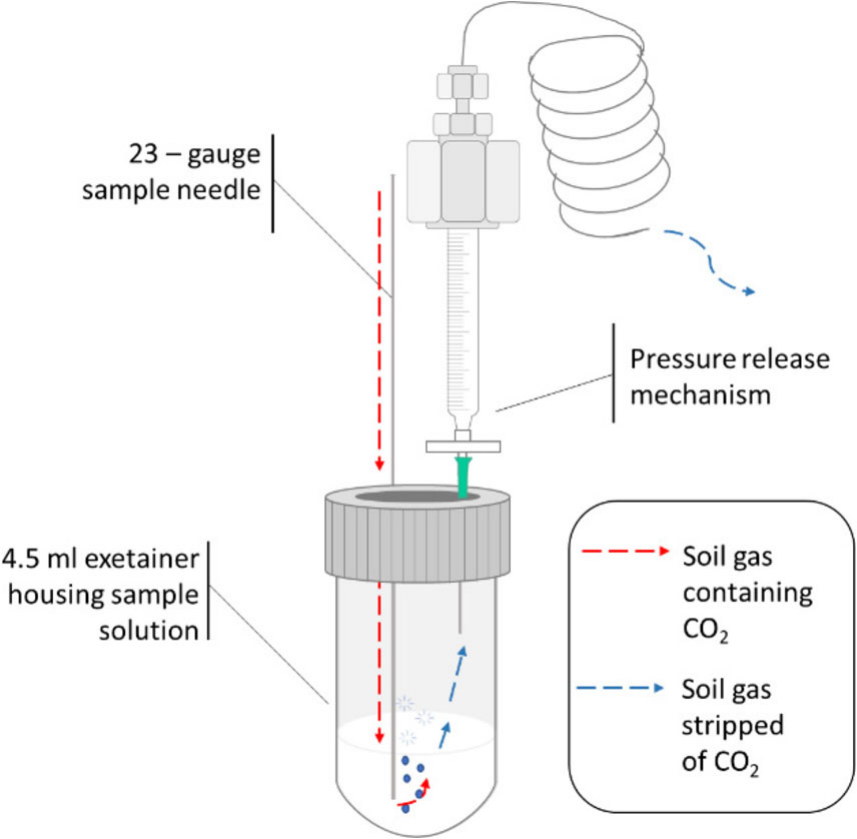
Filter cartridge assembly with Ba(OH)2 for collection CO_2_ from soil gas. Schematic by L. (Reynolds) Shaw [30]

Contamination by a spill or leakage of petroleum hydrocarbons is a significant cause for concern, due to the toxic nature of these fluids and their ability to contaminate not only the soil in which they rest, but also the groundwater and atmosphere at significant distance from the site. Where sites are accessible, active remediation processes can be used, e.g. by trapping with absorbents or using chemicals to oxidize the hydrocarbons to more benign methane or CO_2_. However, a significant number of contaminated sites occur in remote locations where such measures are not practical and so the clean-up must rely on natural source zone depletion. This uses the naturally occurring bacteria to break down the hydrocarbons into CO_2_, but this approach requires a schedule of monitoring the carbon isotope ratio of the CO_2_ released with other soil gases. Some modern CO_2_ is expected from any natural site from root respiration and the degradation of other soil organics, whereas the degradation of the hydrocarbons would produce CO_2_ with no ^14^C because of their age. To analyse a soil gas sample directly requires a large gas volume, an expensive and cumbersome collection, handling and shipping process, as well as further processing by the AMS lab. An alternative sampling method has been developed by Reynolds [30], in which the soil gas is passed through a filter bed of Ba(OH)_2_, as shown in Fig. 5. The CO_2_ reacts with the Ba(OH)_2_ and produces BaCO_3_, while the air and other gases are released. Filter cartridges are simply loaded with Ba(OH)_2_ in a local lab and are used by commercial operators to test remediation sites. Exposed filter cartridges are economically returned to the AMS lab and can be analysed either by directly loading the BaCO_3_ into the ion source or by using an automated Carbonate Handling System connected directly to the AMS ion source.

## Summary

For almost 45 years, AMS has provided highly sensitive isotope analyses for Earth, environmental, archaeometric, bio-medical and materials sciences. New AMS systems and techniques are being developed which require less energy, space and sample preparation time. Applications continue to be developed which are making an impact on research that is important for the sustainability of our society.

## Abbreviations

AMS: Accelerator Mass Spectrometry
B.P.: Before present
CSRA: Compound Specific Radiocarbon Analysis
ECR: Electron Cyclotron Resonance
IRMS: Isotope Ratio Mass Spectrometry
RFQ: Radio-frequency Quadrupole
